# Long-term survival from adenocarcinoma of the esophagus after transthoracic and transhiatal esophagectomy

**DOI:** 10.1186/1477-7819-10-130

**Published:** 2012-06-30

**Authors:** Kjell K Ovrebo, Stein A Lie, Ole D Laerum, Knut Svanes, Asgaut Viste

**Affiliations:** 1 Department of Surgery, Haukeland University Hospital, Bergen, 5021, Norway; 2 Department of Surgical Sciences, University of Bergen, Bergen, Norway; 3 Uni Health, Uni Research, Christies Street 13, Bergen, 5015, Norway; 4 Department of Pathology, The Gade Institute, Section of Pathology, University of Bergen, Haukeland University Hospital, Bergen, Norway

## Abstract

**Background:**

The effects of transthoracic or transhiatal esophagectomy on the long-term survival of patients who had adenocarcinoma of the esophagus were compared, as were factors applicable in preoperative stratification of patient treatment.

**Methods:**

A cohort of 147 consecutive patients with adenocarcinoma of the esophagus was evaluated for esophagectomy between 1984 and 2000. The patients were followed prospectively and observed survival rates of patients with a transthoracic or transhiatal approach to esophagectomy were compared by standardized mortality ratio (SMR) and relative mortality ratio (RMR) using the expected survival of a matched Norwegian population.

**Results:**

A R0 resection was performed by transthoracic (*n =* 33) or a transhiatal (*n =* 55) esophagectomy in 88 (60%) patients with a median age of 61 (range: 35–77) and 70 (42–88) years, respectively (*P <*0.001). Tumor stages and other possible risk factors were similar in the two groups. Transthoracic or transhiatal esophagectomy resulted in a median survival time of 20.5 (95% confidence interval (CI): 10.4–57.6) and 16.4 (10.6–28.7) months, respectively. The respective survival rates were 31.2% and 27.8% by 5 years, and 21.3% and 16.6% by 10 years with an overall RMR of 1.14 (*P =* 0.63). Median survival time in the absence or presence of lymph node metastases was 74.0 (95% CI: 17.5–166.4) and 10.7 (7.9–14.9) months. The corresponding survival rates by 10 years with non-involved or involved nodes were 48.9% and 3.8% respectively (RMR 2.22, *P =* 0.007). Patients with a pT1-tumor were few and the survival rate was not very different from that of the general population (SMR = 1.7, 95% CI: 0.7–4.1). The median survival time of patients with a pT2-tumor was 30.4 (95% CI: 9.0–142) months and with a pT3-tumor 14 (9.2–16.4) months. The survival rates by 10 years among patients with a pT1 tumor were 57.0% (95% CI: 14.9–78.9), pT2 33.3% (11.8–52.2), and pT3 7.1% (1.9–15.5). The relative mortality for T3 stages compared to T1 stages was statistically significant (RMR = 3.22, *P =* 0.024).

**Conclusion:**

Transthoracic and transhiatal esophagectomy are both effective approaches for treatment of adenocarcinoma of the esophagus and survival of more than 10 years can be expected without adjuvant chemotherapy. However, increasing depth of tumor invasion and lymph node metastases reduce life expectancy.

## Background

Adenocarcinoma of the lower esophagus and the gastro-esophageal (GE) junction represent an increasing health problem. In 2005 there were on a worldwide basis nearly half a million new cases and, due to an extremely bad prognosis, almost as many deaths. Once an invasive adenocarcinoma has developed, the diagnosis is usually made at a stage where the disease is already advanced. In Europe and the USA there has been an almost six-fold increase in the incidence of these tumors from 1975 to 2000, and this increase seems to continue [1,2].

Although surgery dominates the treatment of local disease, comparative studies between different surgical approaches are few and controversies still exists on the extent of esophagus resection, lymph node dissection and preferred fields of dissection [3]. Transthoracic esophagectomy is reported with superior survival rates compared with transhiatal esophagectomy [4-7], but the opposite has also been reported [8]. A Dutch trial could not identify any statistical significant difference between the survival rates of patients subject to transthoracic esophagectomy with extended en bloc lymph node resection or transhiatal esophagectomy with limited abdominal lymph node dissection [9,10]. Other reports indicate that gastrectomy with partial esophagectomy and transhiatal esophagectomy for cardiac cancers obtain similar survival rates [11-14]. Minimal invasive approaches to esophagectomy have increased exponentially in England, but mortality rates and length of stay is similar to that of patients treated with conventional surgery [15].

The precision in localization of adenocarcinoma in the distal esophagus may vary and reports on epidemiology and surgical treatment are therefore difficult to evaluate. The Siewert’s classification improved the differentiation and the precision in reports on these tumors and the classification may serve as a base for selection among different surgical approaches [16]. Similar support for treatment algorithms are not necessarily found in the seventh edition of TNM classification [17]. Most studies, however, include patients with both Siewert type I and type II cardiac cancers. When focusing on type I cardiac or distal esophagus adenocarcinoma, survival rates obtained by transthoracic esophagectomy appear superior to that by transhiatal esophagectomy [10]. The diversity of surgical approaches and survival benefit of patients subjected to esophagectomy for adenocarcinoma of the distal esophagus suggest that further studies are needed in order to establish the preferred surgical approach before the addition of different types of multimodal therapy confound evaluation of the surgical approaches.

This study was performed in order to compare the long-term results of transthoracic or transhiatal esophagectomy among patients with type I adenocarcinoma of the esophagus. These patients were all operated before the introduction of multimodal radiochemotherapy.

In order to adjust for all possible demographic confounders that could distort the results and the survival analyses, patient survival was compared with and adjusted for population mortality rates.

## Methods

In the period 1984–2000, 147 consecutive patients with adenocarcinoma of the distal esophagus were evaluated for surgery in this referral center for patients with carcinoma of the esophagus in Western Norway. Patients were registered and followed prospectively.

Distant metastases or poor health obviated a curative resection in 33 (22%) of the patients. Another 26 (18%) patients received an esophagectomy but distant metastases or involved margins of a locally advanced tumor were identified late during the operation implying residual tumor tissue in the patient after resection (R2-resection). This is a prospective study of the remaining 88 (60%) patients subjected to a R0 resection for adenocarcinoma of the esophagus. The patients were not offered adjuvant or neoadjuvant radiochemotherapy. The median time of postoperative observation was 16 (range 0–203) months. Patients subject to a transthoracic or a transhiatal resection were observed for a median of 20 (range 3–203) and 16 (range 0–157) months, respectively.

### Staging

All patients were evaluated by endoscopy. Tumors at or close to the GE junction were classified as adenocarcinoma of the esophagus if the tumor was not visible at the cardia with the endoscope looped or inverted in the stomach. Siewert’s classification [16] of tumors at the GE junction has been introduced, but patients are still offered esophagectomy for tumors invisible at the cardia when inspected from the stomach with a looped endoscope. Preoperative endoscopic ultrasonography (EUS) was introduced for staging of all patients with carcinoma of the esophagus in this department from 1991 [18]. Computed tomography (CT) of the chest and abdomen as well as ultrasonography of the liver and chest radiographs were used for identification of distant metastases. Invasion of the tumor into adjacent organs (Stage III, T4, any N, any M) or distant metastases (Stage IV B) precluded surgery. Surgery was also prohibited among patients with severe symptomatic heart or lung disease, poor general health or cerebral dysfunction.

### Surgical procedures

Esophagectomy was performed with a transhiatal or a transthoracic approach. The surgical approach was determined by patient comorbidity and the surgeons preferred technique.

The transhiatal approach was performed according to Orringer [5,19] with an upper abdominal midline and a left cervical incision. A generous incision of the esophageal hiatus allowed dissection in the lower mediastinum with circumferential removal of the fat pad in the lower mediastinum followed by blunt dissection of the oral esophagus from the esophageal hiatus and thoracic inlet [5,19].

The transthoracic procedure was performed with the patient in a left lateral position, and a right postero-lateral thoracotomy in the fifth or sixth intercostal space. Dissection of the esophagus in the thorax involved en bloc removal of the esophagus, with covering pleura, and all tissue surrounding the esophagus according to Akiyama [20]. The aorta, tracheal membrane and the pericardia served as margin for the dissection and the contralateral pleura was resected as left lateral margin caudal to the pulmonary veins. The azygos vein was routinely divided in the upper mediastinum, but preserved as the dissection was carried out medial to the ascending vein. The thoracic duct was routinely dissected, ligated, and divided at the level of the diaphragm. Lymph nodes in the aorta-pulmonary window were dissected. The bronchial branches of the vagal nerve were preserved. The esophagus was divided orally to the crossing azygos vein and the oral part of the esophagus was dissected into the neck. The patient was later rotated into a supine position for the abdominal and neck dissections.

Both the transhiatal and the transthoracic approach involved extensive lymph node dissection in the upper abdomen and in the lower mediastinum. Tissue and lymph nodes along the common hepatic artery, the celiac trunk and along the top of the pancreas and splenetic artery to the spleen were removed en bloc with lymph nodes along the lesser curve of the stomach, the cardia and the specimen. Lymph nodes were not dissected in the neck.

The left gastric artery was divided at the celiac trunk. The right epiploic arcade and the right gastric artery were carefully preserved. The spleen was removed in patients operated during the 1980s but preserved during the last decade unless its removal was dictated by bleeding.

The esophagus was substituted by a stomach tube in 83 cases. The tube was approximately 5 cm in diameter and created prior to lymph node dissection, by firing a linear stapler several times along the greater curve of the stomach from the angle at the lesser curve to the top of the fundus. Pyloroplasty was not performed routinely, but a Kocher’s maneuver was added in order to improve the reach of the tube. The left colon was used as esophageal substitute in five patients due to former gastric surgery. In all cases the esophageal substitute was pulled up through the posterior mediastinum. The cervical esophagus was approached from the left side of the neck for the anastomosis in all but two patients in whom the anastomosis was performed within the thorax. The cervical anastomosis was constructed with a two-layer Vicryl 3-0, (Ethicon, Norderstedt, Germany) interrupted stitches, a running PDS 4-0 (Ethicon, Norderstedt, Germany) single-layer technique or a circular stapling instrument according to the individual operator’s preferences.

### Postoperative follow up

The patients were observed in a recovery unit until ventilation was adequate and thereafter in a regular ward. The pleural tubes were removed when fluid leakage was less than 100 ml per day. Enteral nutrition was supplied by a transabdominal jejunal catheter from day one and the patients were allowed oral feeding according to their own preferences. Water-soluble oral contrast study (Omnipaque) or endoscopy was performed on suspicion of dehiscence of the anastomosis.

The specimens entered a routine formaldehyde fixation, hematoxylin staining and histopathological evaluation at the department of pathology. No special fat clearance or staining techniques were employed. The stage of disease was classified according to the Union for International Cancer Control’s TNM classification of malignant tumors in the esophagus (sixth edition) [21].

The patients were followed prospectively at the outpatient clinic every 6 months for 2 years, and then once a year for 5 years or until death intervened. Patients referred from other hospitals were followed at their respective hospitals. A few patients were accepted for palliative chemotherapy upon recurrence of disease.

The project was approved by the Regional Committees for Medical and Health Research Ethics (REK 053228) and in addition by the Norwegian Directorate of Health according to the national legislation for biobanks. The investigation was performed according to the World Medical Association Declaration of Helsinki and approved by the Norwegian Social Science Data Services.

### Statistics

The SPSS statistical package version 17 (SPSS Inc, Chicago, USA) was used for the descriptive analyses. The independent sample *t*-tests assessed differences between groups, and non-parametric Mann-tests (Whitney U-test) were used when outliers affected the analyses significantly. Chi-square tests were used to compare categorical data or Fisher exact test when the expected number in any cell of the contingency tables was less than five.

Observed patient survival rates were obtained by the Kaplan–Meier method and presented at 5 (S_O_5) and 10 (S_O_10) years with 95% confidence limits. Median time of survival was calculated and presented with 95% confidence interval (CI). The observed survival curves were compared with expected survival curves calculated from mortality rates, available in 1-year intervals (age and calendar year) for both genders and all birth cohorts, obtained from Statistics Norway [22]. The expected curves reflect thus the survival curves of the Norwegian population with the corresponding composition of gender, age, and year of birth to that of the study group (Expected number of deaths, Exp) as the patients [23].

The standardized mortality ratio (SMR) was used to compare the observed to the expected mortality. The SMR is the ratio of the observed patient mortality and the mortality in the Norwegian population with the same composition of gender, age, and year of birth as that of the study group [24].

In order to compare the SMR of the different covariates (gender, age, stage of disease, surgical approach) a modified Cox-model for time-dependent covariates was fitted [25].

This model includes population mortality rates and adjustment for potential confounders (such as gender, age, stage of disease, surgical approach). The quantities estimated in this model are referred to as relative mortality ratios (RMR). The RMRs (including the 95% CIs) compares the SMRs for the different levels of the variables. In an unadjusted model, the RMR will be almost identical to the SMR of one of the covariates divided by the SMR of the reference category (e.g. RMR_Gender_ = SMR_Female_/SMR_Male_). The analyses were performed with custom and pre-made Fortran programs [23,25]. Results are presented as means or median, and with 95% CIs or range according to appropriate parametric or non-parametric analyses. A *P* value of less than 0.05 was considered statistically significant.

## Results

The median age of the 88 patients with a R0 resection was 67 (range: 35–88) years and the median age of patients with transthoracic (*n =* 33) or transhiatal (*n =* 55) resection was 61 (range: 35–77) and 70 (42–88) years, respectively (*P <*0.001). Men and women were represented in both treatment groups, but males (75) outnumbered females (13) in this series of patients. Clinically important comorbidity was present among 36% of the patients. Table 1 summarizes the characteristics of the patients in the two treatment groups.

**Table 1:** Characteristics of patients subjected to esophagectomy (R0–resections) for adenocarcinoma of the esophagus

	**All**	**Transthoracic**	**Transhiatal**	** *P* **
	** *n =* ** **88**	** *n =* ** **33**	** *n =* ** **55**	
Gender				
females:males	13:75	7:26	6:49	0.222
Age (years)	67 (35–88)	61 (35–77)	70 (42–88)	0.001
Observation (months)	16 (0–203)	20 (3–203)	16 (0–157)	0.179
Comorbidity				
Cardiac	18 (20)	4 (12)	14 (25)	0.177
Pulmonary	12 (14)	2 (6)	10 (18)	0.195
Diabetes mellitus	7 (8)	2 (6)	5 (9)	0.698
Total	32 (36)	9 (27)	23 (42)	0.252

Median and range. Statistics by Fishers Exact Test and Mann-Whitney U-test.

Of the patients with a transthoracic procedure 64% experienced complications and 51% of patients with a transhiatal procedure experienced complications (*P =* 0.152), and some patients experienced several complications (Table 2). Adverse events during the surgical procedure occurred among 13% of the patients. Postoperative complications were dominated by infectious diseases (43%), of which pneumonia (34%) was most frequent; 14% of the patients were predisposed to respiratory failure and ventilator assistance. Clinically important leakage of the anastomosis or esophageal substitute occurred in 6% of the patients but was healed by drainage alone in all patients. Cardiovascular events, thrombosis and embolic complications were also observed. Five patients died in hospital following the transhiatal procedure (9.1%). Two of the five patients were older than 80 years and four of the patients had symptomatic comorbidity. No surgery-related deaths occurred following the transthoracic procedure resulting in an overall in-hospital mortality rate of 5.7%. The median stay in the intensive care unit beyond the recovery period was 1 day and the hospital stay of patients with a transthoracic or transhiatal procedure was 17 days (range 10–114) and 15 days (range 3–256), respectively.

**Table 2:** Surgical events and complications of surgery for adenocarcinoma of the esophagus

	**All resections**	**Transthoracic**	**Transhiatal**	
	** *n =* 88 (%)**	** *n =* 33 (%)**	** *n =* 55 (%)**	** *P* **
Surgical events	11 (13)	6 (18)	5 (9)	0.318
- Recurrent nerve injury	5 (6)	3 (9)	2 (4)	
- Chylous leakage	4 (5)	2 (6)	2 (4)	
- Tracheal injury	2 (2)	1 (3)	1 (2)	
Splenectomy	16 (18)	4 (12)	12 (22)	0.197
Postoperative events				
Pulmonary failure	12 (14)	6 (18)	6 (11)	0.354
Cardiovascular events	5 (6)	0	5 (9)	0.152
Thromboembolism	6 (7)	4 (12)	2 (3)	0.192
Anastomose leakage	5 (6)	2 (6)	3 (6)	0.905
Infectious complications	38 (43)	18 (55)	20 (36)	0.121
- Pneumonia	30(34)	13 (39)	17 (32)	
- Incision	4(5)	3 (9)	1 (2)	
- Abdominal abscess	3(3)	1 (3)	2 (4)	
- Lung abscess	1(1)	1 (3)	0	
Other / minor complications	12 (14)	6 (18)	6 (11)	0.354
Patients with complications	49 (56)	21 (64)	28 (51)	0.275
Intensive care, days (range)	1 (0–69)	1 (0–69)	1 (0–65)	0.457 ^a^
Hospital stay, days (range)	16 (3–256)	17 (10–114)	15 (3–256)	0.086 ^a^
Mortality (in-hospital)	5 (5.7)	0	5 (9.1)	0.152

^a^ Mann–Whitney U test otherwise statistics with Fisher’s exact test.

The average number of retrieved lymph nodes from the specimens was 15.6 (95% CI: 13.5–17.6) and varied between 1 and 49. After transthoracic or transhiatal esophagectomy, the mean number of examined lymph nodes was 17.8 (95% CI: 14.6–21.1) and 14.2 (11.5–16.9), respectively (*P =* 0.089). Patients with lymph node metastases had 1 through 19 involved lymph nodes, and the mean number of involved lymph nodes was 5.9 (95% CI: 3.5–8.2) after transthoracic and 4.1 (2.9–5.3) after transhiatal esophagectomy (*P =*0.184).

The depths of tumor invasion and the frequency of lymph node metastases were similar in the two treatment groups. Approximately 60% of the patients presented with a pT3 tumor or lymph node metastases (Table 3). Lymph node metastases were identified in patients with pT1 tumors (15%) although the frequency was significantly less than among patients with a pT2 (59%) or pT3 tumor (74%) (*P <*0.02) (Table 4).

**Table 3:** Tumor and lymph node stages among patients with a transthoracic or transhiatal esophagectomy for adenocarcinoma of the esophagus

		**Transthoracic**	**Transhiatal**	
	**Total (%)**	** *n =* 33 (%)**	** *n =* 55 (%)**	** *P* **
pT 1	13 (15)	3 (9)	10 (18)	
pT 2	22 (25)	12 (36)	10 (18)	0.125
pT 3	53 (60)	18 (55)	35 (64)	
pN0	34 (39)	13 (39)	21 (38)	1.0
pN1	54 (61)	20 (61)	34 (62)	

Stages based on microscopy of the specimens. Statistics by Fisher’s exact test.

**Table 4:** The frequency of lymph node metastases according to the tumors depth of invasion into the esophagus wall

		**All resections**		
**T-stage**	**no**	**pN 0 (%)**	**pN 1 (%)**	** *P* **
pT 1	13	11 (85)	2 (15) *	*P* <0.02
pT 2	22	9 (41)	13 (59)	
pT 3	53	14 (26)	39 (74)	

* *P* <0.02, pT1 different from pT2 and pT3 by Fisher’s exact test.

**Figure 1:**
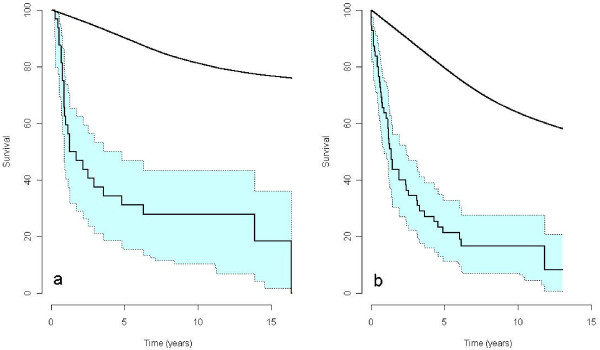
**The observed mortality rate with 95% confidence interval of patients with adenocarcinoma of the esophagus after (a) transthoracic- or (b) transhiatal esophagectomy.** The corresponding mortality rate of the general population with the same composition of age, gender, and year of birth as the treatment group is presented as the uppermost single plot in each panel.

The survival rates after transthoracic or transhiatal esophagectomy were respectively 31.2% and 27.8% by 5 years, and 21.3% and 16.6% by 10 years, and the median time of survival after transthoracic or transhiatal esophagectomy was 20.5 months (95% CI: 10.4–57.6) and 16.4 months (95% CI: 10.6–28.7), respectively. The treatment groups’ similar mortality ratio, indicate that there is no survival benefit from transthoracic esophagectomy when compared with survival rates of transhiatal esophagectomy (RMR = 1.14, *P =* 0.63). Figure 1 and Table 5 illustrate and summarize the statistics in detail.

**Table 5:** Survival rates according to operative procedure, lymph node metastases and primary tumor infiltration in patients treated for adenocarcinoma of the esophagus

	**N**	**Observed deaths**	**S**_**O**_**(5)**	**95% CI**	**S**_**O**_**(10)**	**95% CI**	**SMR**	**95% CI**	**RMR**	**95% CI**	** *P* value**
Total	88	72	25.0	16.2–34.4	20.8	11.8–29.9	6.3	5.0–8.0			
Transthoracic	33	26	31.2	15.5–46.8	27.8	10.4–43.2	6.2	4.2–9.1	1	ref	ref
Transhiatal	55	46	21.3	11.5–32.7	16.6	7.2–27.6	6.4	4.8–8.5	1.14	0.67–1.96	0.63
pN0	34	20	52.6	34.0–67.5	48.9	27.2–64.2	2.8	1.8–4.3	1	ref	ref
pN1	54	52	7.5	2.2–15.9	3.8	0.5–10.0	12.3	9.4–16.2	2.22	1.24–3.98	0.007
pT1	13	5	68.4	35.6–86.2	57.0	14.9–78.9	1.7	0.7–4.1	1	ref	ref
pT2	22	18	38.1	18.0–57.0	33.3	11.8–52.2	3.8	2.4–6.0	1.47	0.49–4.48	0.50
pT3	53	49	9.4	2.9–18.5	7.1	1.9–15.5	13.4	10.1–17.7	3.22	1.16–8.91	0.024
Male	75	62	24.1	15.0–34.2	19.2	10.1–28.9	6.7	5.2–8.6	1	ref	ref
Female	13	10	30.8	6.8–53.7	30.8	3.9–53.7	4.7	2.5–8.7	1.84	0.88–3.86	0.11
<60	28	23	25.0	9.9–41.0	20.8	6.7–36.5	7.9	5.3–11.9			
60–69	23	17	27.3	10.8–45.4	27.3	6.2–45.4	2.4	1.5–3.9			
70–79	28	23	32.1	15.1–48.7	21.4	7.3–38.3	1.5	0.98–2.2			
80+	9	9	0	–	0	–	1.3	0.7–2.4			
Age/10 years									0.43	0.35–0.52	<0.001

S_O_(5) and S_O_(10) are the observed survivals in percent (Kaplan–Meier) at 5 and 10 years respectively with 95% CI. The SMR is the observed mortality (Obs) divided by the mortality in the general population with the same composition of age, gender, and year of birth (Expected number of deaths, Exp) as that of the study group. The RMRs compare the SMRs for different levels of the variables RMR_Gender_ = SMR_Female_/SMR_Male_ , but adjusted for the other factors in the table.

**Figure 2:**
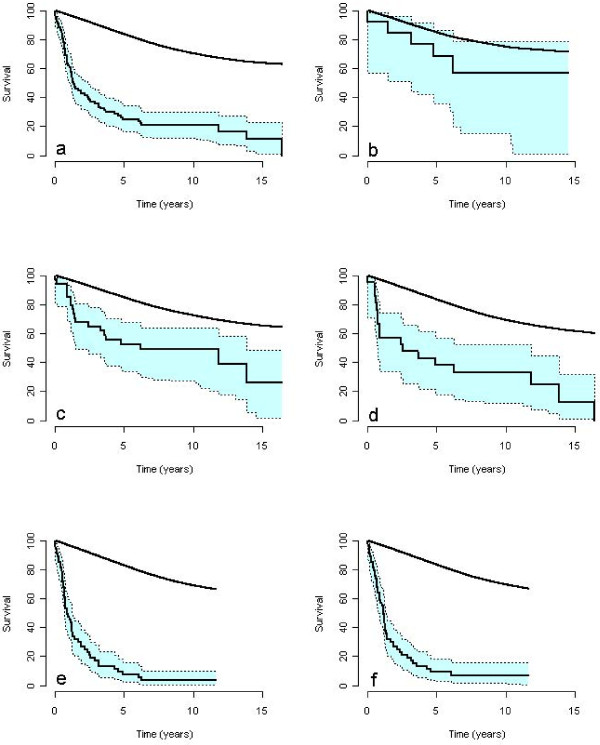
**The observed mortality rate with 95% confidence interval of patients with adenocarcinoma of the esophagus.** The panels illustrate the mortality rate of all patients after esophagectomy **(a)**, patients without lymph node involvement **(c)**, patients with lymph node metastases **(e)**, patients with pT1 tumors **(b)**, patients with pT2 tumors **(d)**, and patients with pT3 tumors **(f)**. The corresponding mortality rate of the general population with the same composition of age, gender, and year of birth as the treatment group is presented in each panel.

The analyses of treatment specific survival rates allowed for pooling of patients in order to study disease and impact of patient specific factors on survival. The overall survival rates of patients after esophagectomy were 25% and 20.8% by 5 and 10 years, respectively with a SMR of 6.3 when compared to the general population (Figure 2a) and the overall median time of survival was 16.4 (95% CI: 12.5–28.7) months.

In the absence of lymph node metastases, the survival rates by 5 and 10 years were 52.6% and 48.9% (Figure 2c), whereas the survival rate was reduced to 7.5% and 3.8% when lymph nodes metastases were identified (Figure 2e) (RMR 2.22, *P =* 0.007, Table 5). Different levels of lymph node involvement were statistically not associated with differences in survival rates of transthoracic or transhiatal esophagectomy in this series of patients. The median times of survival for patients with or without lymph node metastases were 10.7 months (95% CI: 7.9–14.9) and 74.0 months (95% CI: 17.5–166.4), respectively.

The effect of tumor invasion on survival is presented in Figure 2b,d,f. Patients with a pT1 tumor were few and their survival rate was not statistically different to that of the general population (SMR = 1.7, 95% CI: 0.7–4.1) and a median survival time was never reached for this stage of tumor invasion during a maximal follow up of 196 months. Median time of survival for patients with a pT2 tumor was 30.4 (95% CI: 9.0–142) months. The median survival time of patients with a pT3 tumor was 14 (9.2–16.4) months, and the relative mortality of patients with a pT3–stage was statistically increased, when compared with patients with pT1-stage (*P =* 0.024).

Age was a prognostic factor by the time of operation expressed by a relative mortality of 0.43 per 10 years, which implies a decrease in relative mortality for the patients (with respect to the population) of 57% per 10 years of increasing age. The standardized mortality rate was especially pronounced for patients with an age below 70 years (SMR = 2.4) and 60 years (SMR = 7.6) (Table 5). Patients over the age of 80 years did not reach a 5-year survival and their median survival time was 6 months (95% CI: 3.3–8.8).

The patients’ gender was not a prognostic factor for relative mortality in this study.

## Discussion

This study shows that survival of more than ten years can be expected after esophagectomy for adenocarcinoma of the esophagus without adjuvant or neoadjuvant radiochemotherapy. Increasing depth of tumor invasion and the presence of lymph node metastases reduce life expectancy significantly, whereas the surgical approach is less likely to affect the survival rate.

### Method

The statistical methods used in this article adjust for mortality rates from the general population. The analyses hence mimic a study with a (random) control group from the population. By doing this we adjust for the fact that mortality increases with age in the population (and also for the patients) and is higher for males than females. Thus the variables adjusted for in the analyses are also adjusted for the mortality found in the general population [23]. Although the patients are represented in the mortality from the general population, the age-adjusted incidence rates of esophagus carcinoma in Norway is low (2.8–3.4/100,000) [22], and the amount of contribution to the mortality found in the general population is therefore low.

### Morbidity/mortality

Complications are generally observed in 33–71% of patients after esophagectomy [4,9,26], and it is debated whether complication rates are related to type of surgical procedure. Complication rates were statistically not related to surgical procedure in this study. Similar overall morbidity rates of transthoracic (53.5%) and transhiatal (49.3%) esophagectomies are reported by Connors *et al*. [27] after analyzing 17,395 esophagectomies. This is supported by Rentz *et al*. [28] in an analysis of 562 transthoracic and 383 transhiatal resections with a morbidity rate of 47% and 49%, respectively. Only a randomized comparison of transthoracic and transhiatal approaches reveal more chylous leakage, prolonged ventilator support, longer intensive care and hospital stay after transthoracic esophageal resection [9].

The overall mortality rate of 5.7% in this series of patients is similar to that of other reports (2.4–6%) [4,9,26] and higher mortality rates of 8.4–10% or 8.9–9.9% are seen after transthoracic or transhiatal esophagectomy [27,28]. The in-hospital deaths (Table 2) were closely associated with advanced age and comorbidity which is more frequent among patients subject to transhiatal esophagectomy in this series of patients (Table 1).

### Overall survival

The overall SMR of 6.3 relative to the death rate of the general Norwegian population emphasizes the serious prognosis and the vast potential for treatment improvement among patients with adenocarcinoma of the esophagus.

Extensive transthoracic en bloc esophagectomy with a five-year survival rate of 52% [4] is generally considered more efficient and superior to transhiatal esophagectomy with survival rate of 31% [5]. Comparative studies support transthoracic en block resection as a superior procedure to transhiatal resections for all stages of the disease including advanced tumors with limited lymph node metastases [6,7]. Our study shows that any apparent survival benefit of transthoracic over transhiatal surgery is eroded when results are adjusted for year of birth, age, gender, and stage of disease (Table 5). This is in concordance with the Dutch trial that adheres to the Siewert’s classification and includes both type I and type II cardiac cancers [9,10,16]. The Dutch trial show a superiority of transthoracic en bloc resection for type I adenocarcinoma, whereas transthoracic and transhiatal esophagectomy obtain similar survival rates for type II carcinomas [10]. However, the distinction between tumors close to the GE junction is often blurry and lymph node dissection during transhiatal esophagectomy is very different from that of transthoracic esophagectomy in the Dutch study, whereas in our series of patients the lymph node dissection in the abdomen and at the cardia is identical in the two treatment groups.

Moreover, it may be too ambitious to expect any beneficial effect of extended loco regional treatment on patient survival rate when the recurrence rate outside the treatment area is very high. Recurrences after esophagectomy for adenocarcinoma of the esophagus are frequent and approximately 39–56% of recurrences are related to hematogenous dissemination of tumor cells [29-31] of which 37.5% of recurrences occur in the liver, 25% in bones, 17.5% in lung and 11% in the brain [29,32]. The high rates of recurrences outside the dissection area may very well override and extinguish any possible beneficial effects of lymph node dissection by transthoracic or transhiatal esophagectomy on patient survival.

### Survival by stage

The main predictors of outcome in this study, depths of tumor invasion (pT-stage) and lymph node status (pN-stage) (Figure 2, Table 5), are known predictors of local recurrences and distant metastases within one or two years of the operation [4,10,29,30]. Location of lymph node metastases is not predictive of recurrence or survival and therefore not considered in this study [4]. Increasing the number of dissected lymph nodes favorably influences survival rates [33-35]. The varying number of examined lymph nodes in this study may affect precision in staging of patients and distort some of the results in this study by stage migration [35]. In order to improve staging lymph nodes are now harvested from the specimens by the operating surgeon.

Transhiatal esophagectomy is often reported with a more limited lymph node dissection than transthoracic esophagectomy [9,35]. In this study lymph node dissection during transthoracic and transhiatal esophagectomy is identical in the abdomen and at the cardia. Any apparent numeric difference between the two treatment groups is probably related to the additionally dissected lymph nodes in the upper mediastinum during transthoracic esophagectomy.

The complexity of lymph node metastases and outcome by surgical approaches are shown in subanalyses of the Dutch randomized trial [10]. Survival rates of extended transthoracic esophagectomy is superior to that of limited transhiatal esophagectomy when a moderate number of lymph nodes are present (1–8 involved notes), but the survival rates are similar when lymph nodes are uninvolved or massively involved (>8 involved notes) [10]. Similarly Johansson *et al*. [7] show that patients undergoing transthoracic en bloc resection for T3 N1 adenocarcinoma of the distal esophagus had a survival benefit over those treated with transhiatal resection when less than 9 involved lymph nodes are present. Although the lethality among patients with lymph node metastases is high (Figure 2, Table 5), extended mediastinal and abdominal dissection to remove possibly involved nodes is supported by several authors [7,31,32,36].

The depth of tumor invasion is strongly associated with the risk of lymph node metastases (Table 4). Only 15% of patients with a pT1 tumor are at risk of having lymph node metastases. Intramucosal carcinomas are rarely associated with lymph node metastases (8% when confined in lamina propria) (20% when invading the mucosal muscular layer), and limited or even vagal sparing resections are justified in these patients [37]. Locally advanced tumors (pT3) are closely associated with metastases (Table 4) and require more extended surgical options.

It is important to realize that the number of patients eligible for curative resections is limited (60%) and the frequency of regional metastases is high (Tables 3,4). A tailored treatment strategy therefore requires an ambitious preoperative work up to identify patients with possible metastases. Preoperative loco regional staging of esophageal cancer with EUS provides excellent T staging accuracy and the accuracy for N staging compares well with positron emission tomography and CT [38], EUS is also associated with improved survival stratification in patients with esophageal adenocarcinoma [39]. Although the total number of involved lymph nodes is not readily available for preoperative stratification of surgical treatment, the coherence between T stage and N stage seen in this study (Table 4) supports a strategy based on a thorough preoperative evaluation of T stage. We therefore favor T stage and N stage rather than aggregated stages in order to establish a clinical fundament for decision making and comparison in clinical practice.

Patients with adenocarcinoma in the esophagus at an age of less than 60 years have the highest SMRs due to the longer life expectancy of their corresponding matched controls in the background population (Table 5). These patients are therefore likely to benefit the most from multimodal neoadjuvant or adjuvant therapy. The shorter life expectancy of the aged background population explains less SMRs of aged patients. Patients of 80 years or more in this study are prone to in-hospital mortality and a limited length of survival following esophagectomy. This group of patients may therefore benefit from a more palliative approach and esophagectomy may be limited to a selected group of well-functioning patients of 80 years or more.

### Material

The exclusion of patients with residual tumor (R1 or 2) violates the intention to treat principle, but reduces the number of confounding factors in the comparison of transthoracic and transhiatal esophagectomy. Moreover, high-resolution CT, 8F-Fluorodeoxyglucose-positron emission tomography (FDG-PET) and EUS during preoperative work up are now also likely to identify most of these patients prior to surgery [18,40,41]. The allocation of patients to transthoracic or transhiatal esophagectomy is not random and patients with a transhiatal esophagectomy are somewhat older than patients with transthoracic esophagectomy. However, stages of disease (Table 3) are similarly represented in the two treatment groups and the demographic selection biases are adjusted for by statistical methods. Results are therefore considered valid.

### Implications

This study supports both transthoracic and transhiatal esophagectomy as valid options with similar survival and morbidity rates among patients operated without neoadjuvant radiochemotherapy. However, the transthoracic approach is associated with superior survival rates for type I adenocarcinoma in larger series and should probably be the method of choice for patients that can sustain the risk of more morbidity and a longer hospital stay [9,10].

The distinctions in Siewert’s classification are not recognized in later classifications [16,17]. We therefore advocate transthoracic esophagectomy to patients with adenocarcinoma at the distal esophagus that cannot be visualized at the cardia with a looped endoscope.

The work up of these patients is complex and despite an increasing incidence of adenocarcinoma at the distal esophagus and cardia, care for these patients should be confined to a limited number of centers [42].

## Conclusion

Esophagectomy for adenocarcinoma of the esophagus may convey long-term survival of more than ten years without addition of adjuvant therapy. The prognoses are however closely associated with stage of disease and less influenced by surgical approach. The SMR of 6.3 emphasizes a vast potential for improvement of treatment, and strategies for the future should probably involve new multimodal therapy and detection of disease at an early stage.

## Competing interests

The authors declare that they have no competing interests.

## Authors’ contributions

KKO, KS, and AV cooperated in the conception and design of the study and in the collection of the data. ODL validated all pathology reports and SAL assisted in data analysis and interpretation of data. KO drafted the manuscript. All authors read and approved the final manuscript.
